# Genome Sequence of Bluetongue Virus Type 2 from India: Evidence for Reassortment between Outer Capsid Protein Genes

**DOI:** 10.1128/genomeA.00045-15

**Published:** 2015-04-09

**Authors:** Sushila Maan, Narender S. Maan, Manjunatha N. Belaganahalli, Aman Kumar, Kanisht Batra, Pavuluri Panduranga Rao, Divakar Hemadri, Yella Narasimha Reddy, Kalyani Putty, Yadlapati Krishnajyothi, G. Hanmanth Reddy, Karam Pal Singh, Nagendra R. Hegde, Kyriaki Nomikou, Daggupati Sreenivasulu, Peter P. C. Mertens

**Affiliations:** a Vector-borne Diseases Programme, The Pirbright Institute, Pirbright, Woking, Surrey, United Kingdom; b College of Veterinary Sciences, LLR University of Veterinary and Animal Sciences, Hisar, Haryana, India; c Centre for Animal Disease Research and Diagnosis, Pathology Laboratory, Indian Veterinary Research Institute, Izatnagar, India; d Ella foundation, Genome Valley Hyderabad, Telangana, India; e National Institute of Veterinary Epidemiology and Disease Informatics (NIVEDI), Hebbal, Bengaluru, Karnataka, India; f College of Veterinary Science, Sri Venkateswara Veterinary University, Hyderabad, Telangana, India; g Veterinary Biological & Research Institute, Government of Andhra Pradesh, Hyderabad, Telangana, India; h College of Veterinary Science, Sri Venkateswara Veterinary University, Tirupati, Andhra Pradesh, India

## Abstract

Southern Indian isolate IND1994/01 of bluetongue virus serotype 2 (BTV-2), from the Orbivirus Reference Collection at the Pirbright Institute (http://www.reoviridae.org/dsRNA_virus_proteins/ReoID/btv-2.htm#IND1994/01), was sequenced. Its genome segment 6 (Seg-6) [encoding VP5(OCP2)] is identical to that of the Indian BTV-1 isolate (IND2003/05), while Seg-5 and Seg-9 are closely related to isolates from South Africa and the United States, respectively.

## GENOME ANNOUNCEMENT

Bluetongue virus (BTV) is the type species of the genus *Orbivirus*, family *Reoviridae* (1, 2), that exists as at least 27 distinct serotypes (3, 4). The BTV can infect most ruminants, causing severe “bluetongue” disease (BT). The BTV particle is icosahedral and nonenveloped and is composed of a three-layered protein capsid surrounding 10 double-stranded RNA (dsRNA) genome segments ranging in size from 3,944 to 822 bp. The genome segments, identified as segments 1 to 10 (Seg-1 to Seg-10) in order of decreasing size (2), encode 7 structural proteins (VP1 to VP7) and 5 nonstructural proteins (NS1, NS2, NS3/3a, NS4, and NS5) (5–9).

BTV strains from different continents have evolved separately, acquiring multiple point mutations, developing characteristic regional variants/“topotypes” of each genome segment. Full-genome sequence data are available for BTV-2 from Taiwan (10), belonging to the major eastern (e) topotype; a reassortant-strain of BTV-2 carrying a western (w) Seg-5 (11); and five BTV-2(w) isolates (12, 13).

Bluetongue is endemic in India, with evidence for 22 different serotypes (14). A tropical climate (supporting abundant adult Culicoides throughout the year), together with susceptible and high-density sheep populations, all help to maintain the high incidence of BT. Until 1981, clinical BT in India was confined to exotic sheep breeds. However, severe outbreaks subsequently occurred in native Indian breeds (with recorded mortality rates from 2% to 50%, and up to 80% morbidity) in native sheep breeds in Karnataka, Maharashtra, and Andhra Pradesh states, suggesting introduction of exotic BTV strain(s) (14). Nine BTV-serotypes (BTV-1, BTV-2, BTV-3, BTV-9, BTV-10, BTV-12, BTV-16, BTV-21, and BTV-23) have been isolated in India since 2001 (14), with BTV-2 from Maharashtra during 1973 (15), Tamil nadu in 1982, and Andhra Pradesh in 1993, 2007, and 2010 (14).

Indian BTV-2 isolate IND1994/01, from the reference collection (http://www.reoviridae.org/dsRNA_virus_proteins/ReoID/btv-2.htm#IND1994/01) at the Pirbright Laboratory, was grown in BHK-21 cells. Extracted genomic dsRNA was used to synthesize full-length cDNAs by reverse transcription (RT)-PCR, followed by sequencing on a 3730 ABI capillary sequencer using segment-specific primers (16). Seg-1 to Seg-10 of IND1994/01 are 3,944, 2,943, 2,772, 1,981, 1,765, 1,635, 1,154, 1,125, 1,049, and 822 bp, encoding VP1 (1,302 aa), VP2 (962 aa), VP3 (901 aa), VP4 (644 aa), NS1 (552 aa), VP5 (526 aa), VP7 (349 aa), NS2 (354 aa), VP6/NS4 (329/77 aa), NS3/NS3a (229/216 aa), and NS5, respectively.

Phylogenetic analyses show that IND1994/01 contains genome segments derived from both “eastern” and “western” lineages/topotypes. Seg-5/NS1 of IND1994/01 is most closely related (99% nucleotide [nt] identity) to South African isolates (w) (BTV-3 isolate 8231 [JX272593], BTV-3 strain prototype 600565 [FJ713338], and BTV-23 isolate 5268/5 [JX272403]).

Similarly, Seg-9/VP6 shows 99% nt identity with the BTV-10(w) isolate USA/10O80Z [U55781]. Although Seg-2/VP2(OC1) of IND1994/01 shows up to 99% nt identity with other eastern BTV-2 strains, confirming its serotype. Seg-6/VP5(OC2) is identical to Indian BTV-1 strains (IND2003/05), demonstrating reassortment between its two outer-capsid protein genes. It would be useful to investigate the serological characteristics of this virus. Full-genome sequence data for this reassortant BTV-2(r) strain will facilitate investigations of phylogenetic and serological relationships between Indian BTVs.

### Nucleotide sequence accession numbers.

The complete genome sequence of BTV isolate IND1994/01 was deposited in GenBank under the accession numbers KP268774 to KP268783.

## References

[1] Mertens PPC, Maan S, Samuel A, Attoui H 2005 Orbiviruses, Reoviridae, p 466–483. *In* Fauquet CM, Mayo MA, Maniloff J, Desselberger U, Ball LA (ed), Virus taxonomy. Eighth Report of the International Committee on Taxonomy of Viruses Elsevier/Academic Press, London, United Kingdom.

[2] Attoui H, Maan S, Anthony SJ, Mertens PPC 2009 Bluetongue virus, other orbiviruses and other reoviruses: their relationships and taxonomy, p 23–46. *In* Mellor PS, Baylis M, Mertens PPC (ed), Bluetongue Monograph, 1st ed. Elsevier/Academic Press, London, United Kingdom.

[3] Maan S, Maan NS, Nomikou K, Batten C, Antony F, Belaganahalli MN, Samy AM, Reda AA, Al-Rashid SA, El Batel M, Oura CA, Mertens PP 2011 Novel bluetongue virus serotype from Kuwait. Emerg Infect Dis 17:886–889. doi:10.3201/eid1705.101742. 21529403 PMC3321788

[4] Zientara S, Sailleau C, Viarouge C, Höper D, Beer M, Jenckel M, Hoffmann B, Romey A, Bakkali-Kassimi L, Fablet A, Vitour D, Bréard E 2014 Novel bluetongue virus in goats, Corsica, France, 2014. Emerg Infect Dis 20:2123–2132. doi:10.3201/eid2012.140924. 25418049 PMC4257820

[5] Roy P 1992 Bluetongue virus proteins. J Gen Virol 73:3051–3064. doi:10.1099/0022-1317-73-12-3051. 1335020

[6] Firth AE 2008 Bioinformatic analysis suggests that the orbivirus VP6 cistron encodes an overlapping gene. Virol J 5:48. doi:10.1186/1743-422X-5-48. 18489030 PMC2373779

[7] Huismans H, Els HJ 1979 Characterization of the tubules associated with the replication of three different orbiviruses. Virology 92:397–406. doi:10.1016/0042-6822(79)90144-2. 218352

[8] Belhouchet M, Mohd Jaafar F, Firth AE, Grimes JM, Mertens PP, Attoui H 2011 Detection of a fourth orbivirus non-structural protein. PLoS One 6:e25697. doi:10.1371/journal.pone.0025697. 22022432 PMC3192121

[9] Firth AE 2014 Mapping overlapping functional elements embedded within the protein-coding regions of RNA viruses. Nucleic Acids Res 42:12425–12439. doi:10.1093/nar/gku981. 25326325 PMC4227794

[10] Lee F, Ting LJ, Lee MS, Chang WM, Wang FI 2011 Genetic analysis of two Taiwanese bluetongue viruses. Vet Microbiol 148:140–149. doi:10.1016/j.vetmic.2010.08.016. 20855174

[11] Maan NS, Maan S, Nomikou K, Guimera M, Pullinger G, Singh KP, Belaganahalli MN, Mertens PP 2012 The genome sequence of bluetongue virus type 2 from India: evidence for reassortment between eastern and western topotype field strains. J Virol 86:5967–5968. doi:10.1128/JVI.00536-12. 22532533 PMC3347281

[12] Caporale M, Wash R, Pini A, Savini G, Franchi P, Golder M, Patterson-Kane J, Mertens P, Di Gialleonardo L, Armillotta G, Lelli R, Kellam P, Palmarini M 2011 Determinants of bluetongue virus virulence in murine models of disease. J Virol 85:11479–11489. doi:10.1128/JVI.05226-11. 21865388 PMC3194974

[13] Maan NS, Maan S, Guimera M, Pullinger G, Singh KP, Nomikou K, Belaganahalli MN, Mertens PP 2012 Complete genome sequence of an isolate of bluetongue virus serotype 2, demonstrating circulation of a western topotype in southern India. J Virol 86:5404–5405. doi:10.1128/JVI.00420-12. 22492927 PMC3347375

[14] Rao PP, Hegde NR, Reddy YN, Krishnajyothi Y, Reddy YV, Susmitha B, Gollapalli SR, Putty K, Reddy GH 2014 8 28 Epidemiology of bluetongue in India. Transbound Emerg Dis. doi:10.1111/tbed.12258. 25164573

[15] Prasad G, Jain NC, Gupta Y 1992 Bluetongue virus infection in India: a review. Rev Sci Tech 11:699–711. 1335304 10.20506/rst.11.3.624

[16] Maan S, Rao S, Maan NS, Anthony SJ, Attoui H, Samuel AR, Mertens PP 2007 Rapid cDNA synthesis and sequencing techniques for the genetic study of bluetongue and other dsRNA viruses. J Virol Methods 143:132–139. doi:10.1016/j.jviromet.2007.02.016. 17433453

